# Determinants of two-year mortality among HIV positive patients with Cryptococcal meningitis initiating standard antifungal treatment with or without adjunctive dexamethasone in Uganda

**DOI:** 10.1371/journal.pntd.0008823

**Published:** 2020-11-30

**Authors:** Jonathan Kitonsa, Rebecca Nsubuga, Yunia Mayanja, Julius Kiwanuka, Yofesi Nikweri, Martin Onyango, Zacchaeus Anywaine, Abu-Baker Ggayi, Freddie Mukasa Kibengo, Pontiano Kaleebu, Jeremy Day

**Affiliations:** 1 Medical Research Council / Uganda Virus Research Institute & London School of Hygiene and Tropical Medicine Uganda Research Unit, Entebbe, Uganda; 2 Aids Health Care Foundation / Uganda Cares, Masaka, Uganda; 3 Oxford University Clinical Research Unit, Wellcome Trust Major Overseas Programme Vietnam, Ho Chi Minh City, Vietnam; 4 Nuffield Department of Medicine, Oxford, United Kingdom; Universidad de Antioquia, COLOMBIA

## Abstract

Globally, early initiation of antiretroviral therapy for HIV led to a reduction in the estimated mortality from cryptococcal meningitis (CCM) from 624,700 in 2009 to 181,100 in 2014. However, CCM remains one of the leading causes of mortality among HIV infected patients especially in sub-Saharan Africa where 75% of the deaths occur. Most of the studies evaluating mortality have reported short-term mortality (at or before 10 weeks of therapy). We determined mortality and associated factors among patients treated for CCM in the CryptoDex trial (ISRCTN59144167) in Uganda, and the effect of dexamethasone adjunctive therapy on mortality at two years. We conducted a retrospective cohort study between May 2017 and July 2017 to determine the long term survival (up to 2 years post-randomization) of all patients who had been enrolled into the CryptoDex trial in Uganda. The CryptoDex trial recruited between April 2013 and February 2015. We estimated mortality rates and determined factors affecting mortality at two years using Cox regression. The study followed up 211 participants, 127 (60.2%) of whom were male. Sixteen participants (7.58%) were diagnosed with HIV at the same admission when CCM was diagnosed. By two years following randomization 127 (60%) participants had died, a mortality rate of 67 deaths per 100 person-years. Mortality was associated with Glasgow coma score (GCS) below 15 (adjusted Hazard ratio (aHR) 1.77, 95% CI: 1.02–2.44), p = 0.040; weight (aHR 0.97, per 1 Kg increase; 95% CI: 0.94–0.99), p = 0.003; and presence of convulsions (aHR 2.31, 95% CI: 1.32–4.04), p = 0.004, while dexamethasone use and fungal burden had no effect. Long-term mortality in CCM patients remains high even among patients receiving recommended therapy. Strategies to improve long-term survival in CCM patients are urgently needed, especially targeting those with reduced GCS, low weight, and convulsions.

## Author summary

With early initiation of ART among HIV infected patients, mortality from CCM has significantly gone down. CCM however remains the second highest cause of mortality in HIV, the highest burden being in sub-Saharan Africa where 75% of all deaths occur. Most of the studies evaluating mortality have reported short-term mortality. We determined mortality and associated factors among patients treated for CCM using recommended therapy in the CryptoDex trial (ISRCTN59144167) in Uganda, and the effect of dexamethasone adjunctive therapy on mortality at two years. This was done by looking back at all 211 patients who had previously been enrolled into a trial of treatment for CCM (the CryptoDex trial, which recruited patients between April 2013 and February 2015) and determining their survival up to 2 years after they entered the study. This retrospective cohort study was done between May 2017 and July 2017. 127 (60%) of the patients had died by two years. Those with Glasgow coma score (GCS) below 15, those with lower weight, and those that had convulsions were more likely to die, while dexamethasone use and fungal burden did not affect mortality. Long-term mortality in CCM patients remains high even among patients receiving recommended therapy. Strategies to improve long-term survival in CCM patients are urgently needed, especially targeting those with reduced GCS, low weight, and convulsions.

## Introduction

Improvements in the management of HIV including early initiation of antiretroviral therapy have reduced the incidence of opportunistic infections such as cryptococcal meningitis (CCM) [1]. In 2009, there were an estimated 975,000 cases of CCM; by 2014, this had fallen to approximately 225,000. Similarly, the number of estimated deaths fell from 625,000 in 2009 to 180,000 in 2014 [2, 3]. Unfortunately, as these figures illustrate, the risk of death once CCM develops changed little over those five years. CCM remains one of the leading causes of morbidity and mortality among HIV positive patients, coming second to tuberculosis [2–4], and is responsible for 15% of all AIDS-related deaths globally[3].

The highest burden of morbidity and mortality from CCM occurs in sub-Saharan Africa where there were an estimated 162,500 cases (73% of the global figure) with 135,900 deaths (75% of the global figure) in 2014 [3]. Many of the studies evaluating mortality published so far have included patients treated with best available, rather than guideline-recommended therapy, and few studies report mortality rates beyond the period of consolidation therapy [5–9]. This is relevant, because in many places the only available treatment has been fluconazole, although gold standard treatment has been based around intravenous amphotericin. As such, these studies may miss longer-term mortality effects that could be consequences of initial treatment. For example, different initial treatments may have different effects on the risk of disease relapse and disability, both of which may influence long-term survival [10].

Even so, the mortality on amphotericin-based therapy is high, reported to vary between 20% and 50% by 10 weeks from diagnosis. In addition to treatment, the risk of death has been associated with factors such as elevated intracranial pressure, slower fungal clearance from cerebrospinal fluid, and drug toxicities, amongst others [11–14]. Among the few studies that determined longer-term mortality, Butler K et al found a mortality of 66% at two years from treatment initiation among patients treated for CCM using recommended therapy in Mulago hospital in Uganda. This study explored the effect of only a few variables on mortality and the model included patients with cryptococcal antigenaemia but without CCM [15]. The majority of studies of treatment for CCM follow patients for between 3 and 6 months[14]. Up to this point, determinants of outcome include measures of disease severity at baseline, such as fungal burden (the amount of culturable yeast in cerebrospinal fluid), and treatment choice [14]. However, survival curves suggest that deaths may continue beyond this point for patients who do not receive initial treatment with Flucytosine [14]. We wanted to better understand the long term outcomes of cryptococcal meningitis in our country, and how this is influenced by treatment and non-treatment factors among CCM patients, including those previously associated with short term outcome. Identifying patients at increased risk of late death, while they are still engaged in care, may enable the development of strategies to improve their prognoses. We determined two-year mortality and associated factors (including the effect of dexamethasone adjunctive therapy) among patients previously enrolled in the CryptoDex trial (Clinical trial registration number ISRCTN59144167, PMID: 25391338) [16] in Uganda. The CryptoDex trial was stopped early due to safety concerns [16].

## Methodology

### Ethics statement

The study was approved by the Makerere University School of Public Health Research and Ethics Committee. Permission to use information from the CryptoDex database was acquired following standard MRC/UVRI & LSHTM Uganda Research Unit data sharing policy available at https://www.mrcuganda.org/publications/data-sharing-policy. Written informed consent was obtained from all participants that had been exited from the CryptoDex trial before data collection. For participants that had died, the next of kin provided consent.

### Study design and setting

We conducted a retrospective cohort study among patients previously enrolled in the CryptoDex trial in Uganda. The CryptoDex trial was a double-blind placebo-controlled phase III study of adjunctive dexamethasone in HIV infected adults with Cryptococcal meningitis conducted between April 2013 and February 2015 at thirteen sites in six countries in Asia and Africa. The Ugandan sites included two research centres belonging to the Medical Research Council/Uganda Virus Research Institute and London School of Hygiene and Tropical Medicine Uganda Research Unit (MRC/UVRI & LSHTM Uganda Research Unit), located at Masaka and Entebbe. Entebbe and Masaka are located 37 and 140 kilometres south-west of Kampala city respectively. Participants enrolled at Masaka research centre were admitted to Masaka Regional Referral Hospital and mainly came from rural settings that constitute the hospital catchment area. Participants enrolled at Entebbe research centre were admitted to Entebbe General Hospital and mainly came from semi-urban settings that serve as the catchment area for the hospital.

### CryptoDex subjects, their management and follow up

Participants recruited in the CryptoDex study were 18 years and above, had HIV infection, a clinical syndrome consistent with CCM, i.e., one or more of fever, neck stiffness, severe headache, altered consciousness amongst others, and microbiologically confirmed disease as indicated by one or more of the following results: positive India ink staining of cerebrospinal fluid (CSF), culture of Cryptococcus species from CSF or blood, or cryptococcal antigen detected in CSF on cryptococcal antigen lateral flow assay (IMMY). Other components of the inclusion and exclusion criteria may be accessed in the published trial protocol [16]. Eligible patients were randomised in a 1:1 ratio to either dexamethasone adjunctive therapy or placebo in addition to antifungal treatment with intravenous Amphotericin B (1 mg per kilogram per day) and 800mg daily oral Fluconazole for 14 days (induction phase). This was followed by a continuation phase with 800mg daily fluconazole for eight weeks, and then a maintenance phase with oral fluconazole 200mg daily. At the time, this was the regimen recommended by WHO for settings where Flucytosine was not available [12]. Patients received either dexamethasone or placebo for 6 weeks as follows: intravenous administration of 0.3 mg and 0.2 mg per kilogram of body weight per day during the first and second weeks respectively, followed by oral administration of 0.1 mg per kilogram per day during the third week, 3 mg per day during the fourth week, 2 mg per day during the fifth week, and 1 mg per day during the sixth week. Participants were followed up for six months after enrolment.

### Study subjects and data collection for the current study

This study was conducted between May and July 2017: we collected data on all participants enrolled in the CryptoDex trial in Uganda to establish their survival status by the end of two years from randomisation. Data was collected using semi-structured questionnaires, which were developed in English, translated to Luganda, and back-translated. These were standardised and piloted before use to assess comprehensibility. Trained research assistants administered the questionnaires. Information for participants who died while still in the CryptoDex trial was already available in the study database. Information for those that died after exit was acquired through a next of kin while living participants were contacted in person. Participants were tracked using contact details previously collected while in the CryptoDex trial. One session was organised with each participant or next of kin during which an interview was administered. Data collected was supplemented by that already available in the CryptoDex database.

### Study variables

The outcome variable was death within two years after randomization in the CryptoDex trial. Predictor variables tested included treatment arm (placebo or dexamethasone); demographic characteristics (gender, age, and education measured as number of years in school); site, i.e. Masaka (rural) or Entebbe (semi-urban); ART status, i.e. on or not on ART; previous history of CCM, i.e., ever or never suffered from CCM; history of convulsions; Glasgow coma Score, i.e., 15 or <15; presence or absence of confusion; weight; blindness (No light perception in one or both eyes); blurred vision; hearing impairment (reduced hearing in one or both ears); presence of convulsions (at or before randomisation); first lumbar puncture opening pressure; yeast quantitative count in cerebrospinal fluid (CSF); and white blood cell count in CSF. Variables were chosen based on literature review.

### Data analysis

Data was entered into a Microsoft Access database, cleaned and analysed in STATA (College Station, TX, version 15.0). Frequencies and percentages were used to summarise categorical variables while continuous variables were summarised using means (standard deviation) and / or medians (interquartile ranges (IQR)). Time accrued was measured in person-years, estimated as the difference in time from entry (Randomisation) to death or time of censor (completion of 2 years from randomisation or time last known to be alive). We estimated survival using Kaplan-Meier (K-M) curves for the whole cohort and as per the intervention (Dexamethasone/placebo). We used Cox regression models to estimate overall mortality rate as well as crude and adjusted mortality rates. Variables that had a likelihood ratio test (LRT) p-value of 0.2 and below in the crude analysis were included in the multivariable Cox regression models. Variables were retained in the multivariable model if their inclusion resulted into a better model than the model without the variable based on the LRT p-value (p-value<0.05) using a stepwise building approach. A test of proportionality of hazards was conducted for the variables evaluated individually and for the final model.

## Results

*All patients were treated with 2 weeks of amphotericin B 1mg/kg daily and 2 weeks of fluconazole, 800mg/day followed by 8 weeks of fluconazole 800mg daily, then fluconazole 200mg/day.

### Baseline characteristics

**Fig 1:**
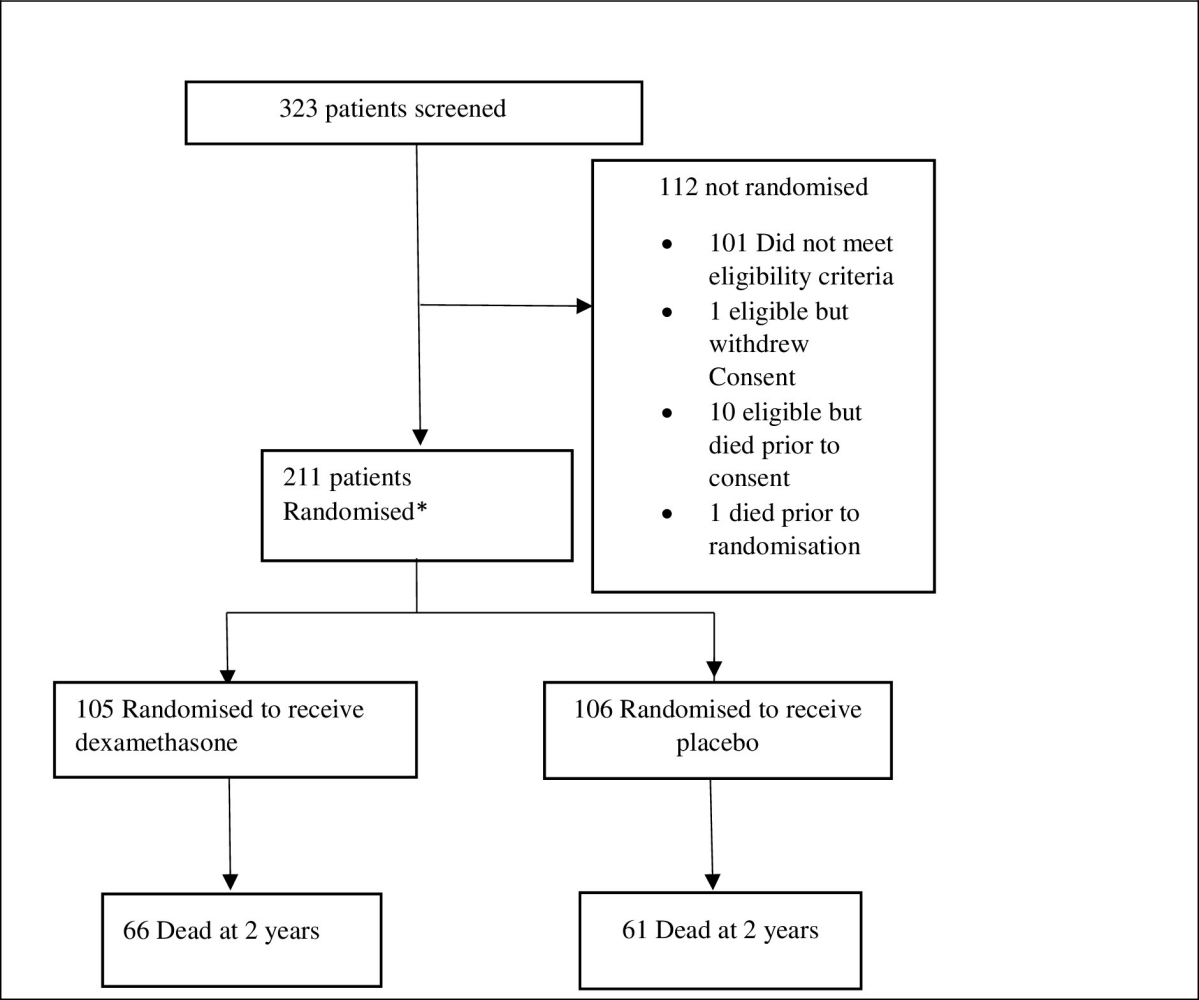
Study flow diagram showing participants enrolled in the CryptoDex trial in Uganda and mortality through two years after initiation of therapy.

Fig 1 is a flow diagram showing participants enrolled in the CryptoDex trial in Uganda and mortality through two years from treatment initiation of therapy. Out of 323 patients screened at the two sites in Uganda, 212 were found eligible for participation in the CryptoDex trial, one died before randomisation, and 211 were randomised. Data for all randomised participants was used in this analysis (Fig 1). Of these, 127 participants (60.2%) were male. The mean age (SD) was 36.4 years (8) and one hundred and five participants (49.8%) were randomised to receive dexamethasone adjunctive therapy. Sixteen patients (7.6%) were diagnosed with HIV on admission with CCM, while 195 (92.4%) were already known to be HIV positive with 116 (59.5%) of these already on ART. The median last CD4 (IQR) done prior to enrolment was 29 (7–71) cells/uL. Of all participants, 131 (65.5%) had CSF opening pressure ≥18cm of CSF, and 118 (55.9%) had more than 1000 yeast cells per ml of CSF. Twenty-five participants (11.8%) reported history of convulsions and fifty (23.7%) had haemoglobin counts below 10mg/dl. The baseline characteristics are summarised in Table 1.

**Table 1:** Baseline characteristics of study participants at randomisation in the CryptoDex trial.

Variable		Treatment				Total 211
		Placebo (N = 106)		Dexamethasone (N = 105)		
	Level	N	%	n	%	
Gender	**Male**	60	56.6	67	63.8	127
	**Female**	46	43.4	38	36.2	84
Age	**18–30 Years**	40	37.7	28	26.7	68
	**31–39 Years**	33	31.1	44	41.9	77
	**40+ Years**	33	31.1	33	31.4	66
Education	**≤7 Years**	65	73.9	52	59.1	117
	**>7 Years**	23	26.1	36	40.9	59
Site	**Entebbe**	43	40.6	43	41.0	86
	**Masaka**	63	59.4	62	59.0	125
HIV diagnosis known before	**No**	10	9.4	6	5.7	16
	**Yes**	96	90.6	99	94.3	195
Previous CCM	**No**	98	92.5	95	90.5	193
	**Yes**	8	7.5	10	9.5	18
History of Convulsion	**No**	90	84.9	95	91.3	185
	**Yes**	16	15.1	9	8.7	25
Glasgow coma Score	**15**	83	78.3	82	78.1	165
	**<15**	23	21.7	23	21.9	46
Confusion	**No**	65	61.9	64	61.9	129
	**Yes**	40	38.1	41	38.1	81
Mean Weight kg (SD)		51.2 (9.59)		50.7 (10.61)		51.0 (10.09)
Blindness*	**No**	96	99.0	96	99.0	192
	**Yes**	1	1.0	1	1.0	2
Blurred vision	**No**	77	79.4	75	77.3	152
	**Yes**	20	20.6	22	22.7	42
Hearing Impairment	**No**	88	88.9	86	86.9	174
	**Yes**	11	11.1	13	13.1	24
CSF Opening Pressure cm/CSF	**≤18**	37	36.6	32	32.3	69
	**>18**	64	63.4	67	67.7	131
Yeast CFUs per ml of CSF	**≤1000**	45	42.5	48	45.7	93
	**>1000**	61	57.5	57	54.3	118
CSF white-cell count cells/mm^3^	**<5**	2	2.0	0	0.0	2
	**≥5**	97	98.0	99	100.0	196
Haemoglobin g/dl	**≥10**	79	74.5	82	78.1	161
	**<10**	27	25.5	23	21.9	50

**Abbreviations:** SD, Standard; HIV, Human immunodeficiency virus; CFUs, Colony-forming units; CCM. Cryptococcal meningitis; g/dl, grams/decilitre.

*No light perception in one or more eyes.

### Mortality by two years

**Fig 2:**
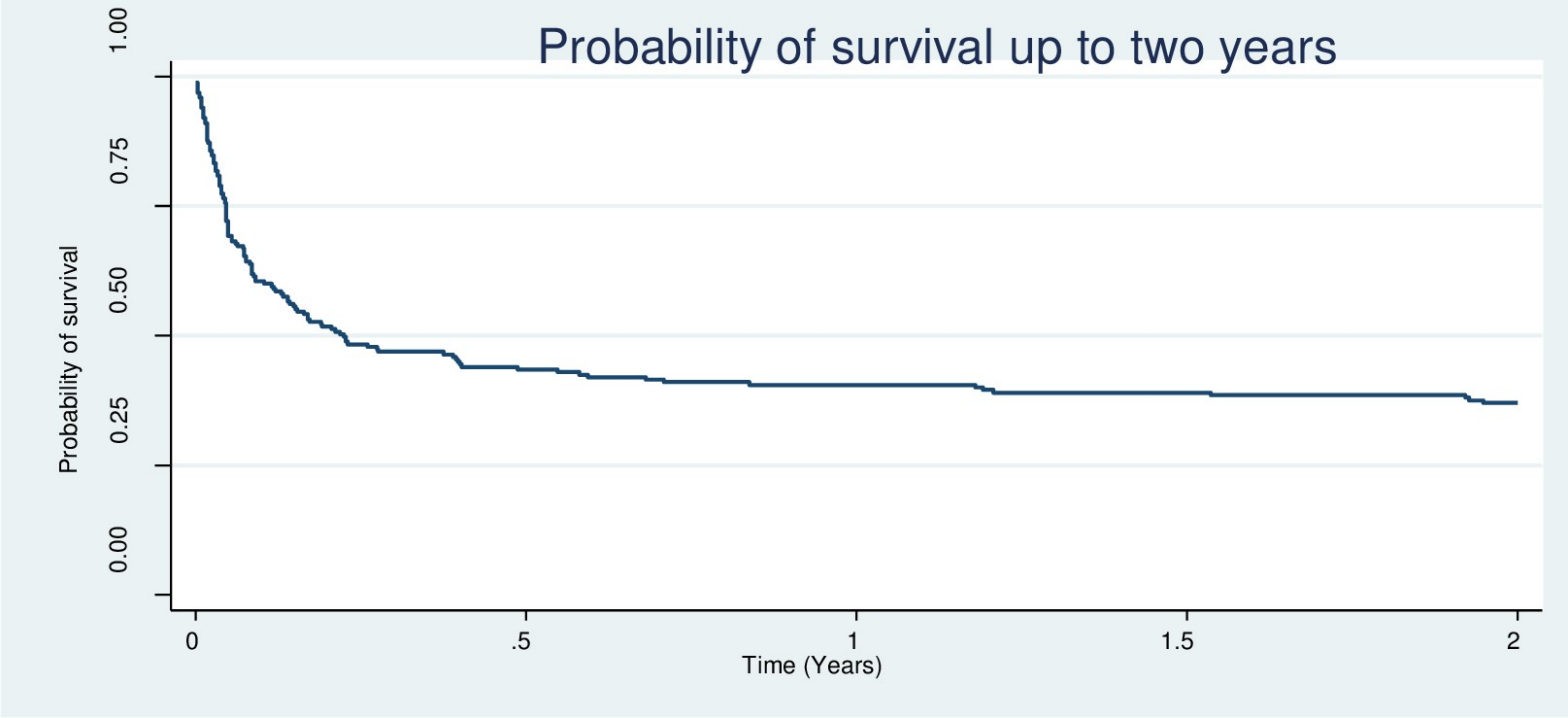
Kaplan-Meier plot showing survival of participants in the first 2 years of enrolment.

**Fig 3:**
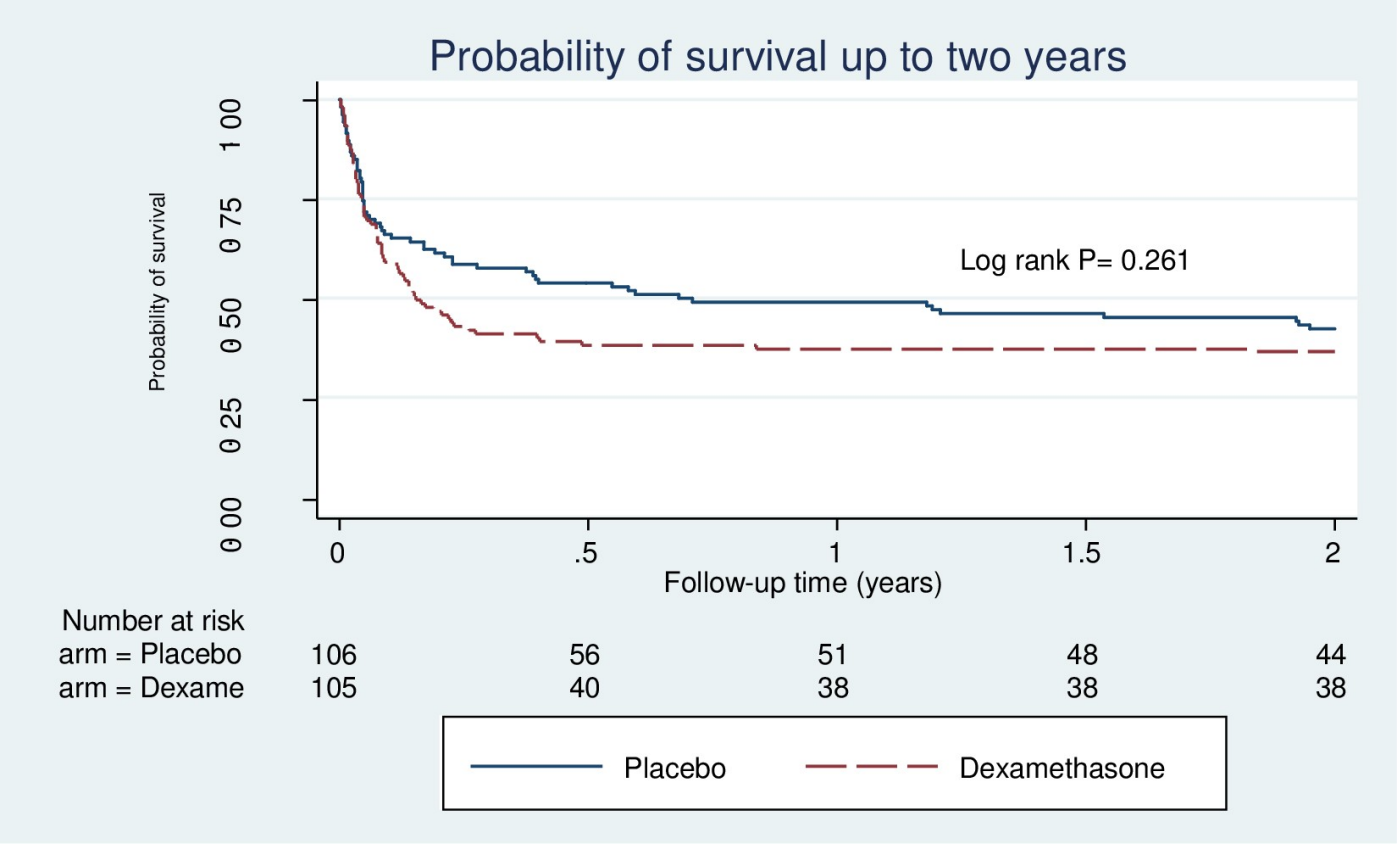
Kaplan-Meier plot showing survival of participants in the first 2 years of enrolment categorised per intervention.

All models assessed passed the test of proportional hazards assumption. At two years from initiation of treatment for CCM, 127 patients had died (proportionate mortality of 60%), giving a mortality rate of 67 deaths per 100 person-years after 190.7 person-years of follow up. The mortality rate was 57 and 79 deaths per 100 person-years among patients that received placebo and dexamethasone adjunctive therapy respectively. The median survival time was approximately 3 months. Basing on the Kaplan-Meier plot (Fig 2), mortality was highest during the first 6 months. Fig 3 shows mortality by intervention.

### Factors associated with mortality

Results from the univariate and multivariate analysis are summarised in Table 2.

**Table 2:** Mortality rates and effect of different factors on mortality at two years.

Variable				Unadjusted		Adjusted*	
	Level	Number of deaths/Person years at risk	Mortality per 100 person-yrs.	HR	P Value	HR(95% CI)	P-value
Overall		127/191	67				
Intervention*	**Placebo**	61/107	57				
	**Dexamethasone**	66/84	79	1.22 (0.86–1.73)	0.264	1.32 (0.91–1.91)	0.139
Gender	**Male**	75/114	66				
	**Female**	52/77	68	1.04 (0.73–1.48)	0.845		
Age	**18–30 years**	38/72	53				
	**31–39 years**	46/64	75	1.25 (0.82–1.92)	0.297		
	**40+ years**	41/55	74	1.29 (0.83–2.01)	0.254		
Education	**≤7 Years**	70/105	67				
	**>7 Years**	33/62	53	0.84 (0.56–1.27)	0.415		
Site*	**Entebbe**	57/69	83				
	**Masaka**	70/122	58	0.76 (0.54–1.08)	0.126	0.69 (0.47–1.00)	0.052
HIV known before Baseline	**No**	7/19	38				
	**Yes**	120/172	70	1.55 (0.72–3.32)	0.259		
On ARVS	**Yes**	66/117	56				
	**No**	61/77	77	1.31(0.92–1.84)	0.133	1.22 (0.84–1.76)	0.298
Previous CCM	**No**	115/175	67				
	**Yes**	12/16	76	1.12 (0.62–2.03)	0.705		
History of Convulsion*	**No**	105/180	58				
	**Yes**	21/11	194	2.32 (1.45–3.71)	<0.001	2.31 (1.32–4.04)	0.004
Glasgow coma Score*	**15**	91/166	55				
	**<15**	36/25	143	1.77 (1.20–2.61)	0.004	1.58 (1.02–2.44)	0.040
Confusion	**No**	73/125	59				
	**Yes**	53/66	81	1.28 (0.90–1.83)	0.166		
Weight* kg				0.96 (0.94–0.98)	<0.001	0.97 (0.94–0.99)	0.003
Blurred vision	**No**	85/150	57				
	**Yes**	26/37	71	1.15 (0.74–1.78)	0.541		
Hearing Impairment	**No**	100/167	60				
	**Yes**	15/21	73	1.11 (0.64–1.91)	0.706		
CSF Opening Pressure* cm/CSF	**≤18**	46/54	85				
	**>18**	74/128	58	0.77 (0.53–1.11)	0.168	0.67 (0.44–1.01)	0.056
Yeast CFUs per ml of CSF	**≤1000**	53/91	58				
	**>1000**	74/100	74	1.20 (0.84–1.70)	0.321		
Haemoglobin* g/dl	**≥10**	90/157	57				
	**10<**	37/33	111	1.51 (1.03–2.22)	0.035	1.20 (0.78–1.84)	0.412

**Abbreviations:** SD, Standard deviation; ART, Antiretroviral therapy; CFUs, Colony-forming units; H2O, Water; CI, Confidence interval; HIV, Human immunodeficiency virus; kg, kilogram; g/dl, grams/decilitre.

*Variables included in the final model.

### Crude analysis

In the univariate analysis, the factors significantly associated with mortality included Glasgow coma score below 15 (HR 1.77, 95% CI: 1.20–2.61), p = 0.004; weight, with the hazard of mortality reducing by a factor of 0.96 for every kilogram increase in weight (HR 0.96, 95% CI: 0.94–0.98), p<0.001; haemoglobin count below 10g/dl (HR 1.51, 95% CI 1.03–2.22), p = 0.035; and presence of convulsions (HR 2.32, 95% CI: 1.45–3.71), p<0.001. There was no association between mortality within two years and dexamethasone use (HR 1.22, 95% CI: 0.86–1.73), p = 0.264, although the CryptoDex study had been stopped early following an interim safety analysis because of evidence of increased rates of adverse events and slower fungal clearance in patients receiving dexamethasone.

### Adjusted analysis

From the multivariate analysis, the factors independently significantly associated with mortality within the first two years following diagnosis were Glasgow coma score below 15 (aHR 1.77, 95% CI: 1.02–2.44), p = 0.04; weight, with the hazard of mortality decreasing by a factor of 0.97 for every kilogram increase in weight (aHR 0.97, 95% CI 0.94–0.99), p = 0.003; and presence of convulsions (aHR 2.31, 95% CI: 1.32–4.04), p = 0.004. There was no relationship between mortality at 2 years and gender, age, ART status at CCM diagnosis, haemoglobin count, or yeast quantitative count in CSF.

## Discussion

By the end of two years of follow-up, 127 of the 211 study participants had died yielding 60% proportionate mortality and an overall mortality rate of 67 deaths per 100 person-years. This mortality rate contrasts markedly with that previously reported for Ugandan HIV patients with CD4 counts similar to those of our patients, i.e., below 50cells/ml, of 6.73 deaths per 100 person-years [17]. However, we believe our finding is plausible since it is consistent with the proportionate mortality of 66% reported by Butler et al from their study of patients with cryptococcal meningitis from Mulago Hospital, Uganda [15].

Apart from cryptococcal meningitis itself, other potential contributors to mortality during this period include drug-related toxicities, immune reconstitution syndrome (IRIS), morbidity associated with residual disability, and other infections, since patients are still severely immune-suppressed [18]. Like others, we found that most deaths did occur within the first 6 months following diagnosis [14, 19]. This suggests that efforts to improve early treatment of cryptococcal disease are likely to deliver the best long-term gains.

Unfortunately, there is a dearth of therapeutic options for cryptococcal meningitis. Cryptococcal meningitis continues to be managed using drugs that were discovered more than 60 years ago, whose use is also associated with multiple toxicities [20], while 30–40% of patients fail to attain CSF sterilization [14, 19, 21]. There has been little real impact on mortality in the last 20 years. Recent efforts examining drug repurposing and adjunctive therapy have been disappointing [18, 22]. We therefore agree with previous researchers that alternative and complementary approaches for the management of CCM still need to be investigated to curb mortality [11, 18]. Flucytosine remains expensive (>$120 per day) and unlicensed in most countries in sub-Saharan Africa including Uganda. The most practical intervention remains early HIV testing and intensive screening for CCM using the available Cryptococcal antigen tests which enable identification of asymptomatic antigenaemia a median of 22 days before meningitis sets in [23, 24]. It has been estimated that screening and pre-emptively treating people with positive antigenaemia in Uganda would cost only 15% of the cost of meningitis treatment, with more than 40% better long-term survival [15].

Mortality was not significantly different between patients treated with adjunctive dexamethasone therapy and those on placebo. This result is similar to findings from the CryptoDex study where no difference in mortality was observed at 10 weeks and 6 months [18]. This is perhaps not surprising given that the CryptoDex study was stopped after the enrolment of 451 patients following an interim safety analysis because of evidence of accumulating harm in patients receiving dexamethasone, in the form of increased rates of adverse events and slower fungal clearance. However, it is reassuring that deaths did not continue to accumulate as a consequence of the receipt of dexamethasone. It is however clear that dexamethasone neither improves short term or longer-term survival from CCM. The 2018 WHO guidelines on cryptococcal treatment recommend against use of corticosteroids during the induction treatment phase for CCM [25].

It had been hypothesised in the CryptoDex study that dexamethasone would improve outcomes by reducing both intracranial pressure and inflammatory complications, and decreasing the incidence of IRIS. Analysis done on CSF from a subset of patients in the CryptoDex study revealed that there was a downregulation of pro-inflammatory cytokines among dexamethasone recipients [26]. This could explain the absence of benefit.

Recently data has emerged from the ACTA trial of combination antifungal therapy for CCM in Africa, suggesting that despite good sterilising power, longer courses of amphotericin (2 versus 1 weeks) may be harmful to patients [27]. In this study, the best outcomes were seen in patients receiving amphotericin for one week, in combination with Flucytosine. Patients receiving two weeks of amphotericin had more adverse events, and worse survival (although the latter did not reach statistical significance). While the sterilising effect of one and two-week amphotericin regimes over the first two weeks seemed similar, it will be important to see whether there is any effect on long term survival. Differences in induction therapy appear to be able to influence long term outcomes, certainly up to 6 months after diagnosis [14].

We found that higher weight was associated with a reduced risk of mortality. Other studies among CCM patients have reported a similar finding, though this was after much shorter periods of follow up [19, 28]. An association between greater weight and improved survival is plausible since it is likely that the lighter patients had more advanced HIV disease and less physiological reserve. Scherzer R et al previously reported that lower muscle mass was associated with increased risk of mortality among HIV patients in general [29].

Presence of convulsions was also associated with increased risk of mortality. A similar finding has previously been reported in previous studies [30, 31]. Convulsions may be associated with more severe disease and/or other effects in the brain that could increase the risk of mortality. The possibility of other disease manifestations such as cryptococcomas, or comorbidities that can cause convulsions cannot be entirely ruled out in our patients because, unfortunately, advanced investigatory modalities like CT scans are not readily available in settings such as our own.

Although not statistically significant, participants from Masaka were less likely to die than those from Entebbe. Masaka is a rural setting, while Entebbe and its catchment area is mainly semi-urban. Possibly, the social set up of people in rural settings in Masaka enabled closer social support by family members for those that were sick or recovering from disease compared to those from Entebbe. Presence of social support has previously been reported to be associated with better health outcomes [32, 33].

We did not find that factors previously reported by others to be associated with short-term mortality such as white blood cell count in CSF, haemoglobin count, age, and altered mental status, affected longer-term mortality in our study [19, 28]. Such associations may diminish in importance as patients survive for longer periods.

We were unable to measure other factors that could have a bearing on long-term mortality such as antifungal treatment resistance. Antifungal susceptibility is considered to be relevant to treatment response in relapsed disease, but susceptibility testing at the point of first diagnosis appears to have little utility; few data exist on which to make a judgement regarding its ability to determine long term outcomes [34]. It would have been good to explore the effect of CD4 counts on mortality. Unfortunately, CD4 counts were not measured at baseline in the CryptoDex study. The requirement was for participants to report their last CD4 counts at enrolment. Many had forgotten/had no record or presented those done a long time prior. It would have also been good to see the effect of baseline viral loads on mortality. These were however not tested in the CryptoDex trial.

## Conclusion

This study shows that long-term mortality in CCM patients, even among those receiving recommended therapy remains high. Adjunctive therapy with dexamethasone does not have a significant effect on mortality at two years, while wasted patients, those who present with impaired consciousness as defined by Glasgow Coma score <15, and those with histories of convulsions, are more likely to die.

We recommend extensive implementation of Cryptococcal screening in low resource settings to prevent CCM whose management remains a daunting and challenging experience. There is a pressing need to develop better treatment for those patients who present with the disease.
